# The importance of understanding individual differences in Down syndrome

**DOI:** 10.12688/f1000research.7506.1

**Published:** 2016-03-23

**Authors:** Annette Karmiloff-Smith, Tamara Al-Janabi, Hana D'Souza, Jurgen Groet, Esha Massand, Kin Mok, Carla Startin, Elizabeth Fisher, John Hardy, Dean Nizetic, Victor Tybulewicz, Andre Strydom

**Affiliations:** 1 Centre for Brain & Cognitive Development, Birkbeck University of London, London, WC1E 7HX, UK; 2 The London Down Syndrome Consortium (LonDownS), University College London, London, UK; 3 Division of Psychiatry, University College London, London, W1T 7NF, UK; 4 The Blizard Institute, Barts & The London School of Medicine, Queen Mary University of London, London, E1 2AT, UK; 5 Department of Molecular Neuroscience, University College London Institute of Neurology, London, WC1N 3BG, UK; 6 Division of Life Science, Hong Kong University of Science and Technology, Hong Kong SAR, China; 7 Department of Neurodegenerative Disease, Institute of Neurology, London, WC1N 3BG, UK; 8 Lee Kong Chian School of Medicine, Nanyang Technological University, Biopolis, 138673, Singapore; 9 Francis Crick Institute, London, NW7 1AA, UK; 10 Department of Medicine, Imperial College London, London, W12 0NN, UK

**Keywords:** Down syndrome, Alzheimer’s disease, neurodevelopmental disorder, trisomy 21

## Abstract

In this article, we first present a summary of the general assumptions about Down syndrome (DS) still to be found in the literature. We go on to show how new research has modified these assumptions, pointing to a wide range of individual differences at every level of description. We argue that, in the context of significant increases in DS life expectancy, a focus on individual differences in trisomy 21 at all levels—genetic, cellular, neural, cognitive, behavioral, and environmental—constitutes one of the best approaches for understanding genotype/phenotype relations in DS and for exploring risk and protective factors for Alzheimer’s disease in this high-risk population.

## Introduction

Down syndrome (DS) is the most common neurodevelopmental disorder of known genetic cause, with an incidence of between 1:750 and 1:1000 live births ^1, 2^. The syndrome has been extensively described at the group level, downplaying individual variation and treating DS as a homogeneous group. So, why do we argue in this paper that individual differences across DS at all levels—genetic, cellular, neural, cognitive, behavioral, and environmental— really matter? Our argument is that, in the context of significant increases in DS life expectancy ^3, 4^, a focus on individual differences in trisomy 21 constitutes one of the best approaches for exploring genotype/phenotype relations in DS and for identifying risk and protective factors for Alzheimer’s disease (AD).

DS has usually been described simply as arising from an extra copy of chromosome 21 and presenting with characteristic features including facial dysmorphology, a proportionally large tongue, low muscle tone, short stature, and intellectual disability. Associated conditions may include obstructive sleep apnea, as well as visual and hearing problems. Receptive language usually outstrips language production, spatial memory is thought to be better than verbal memory, and global processing is deemed to be superior to local processing. In adulthood, DS presents with accelerated aging and an increased likelihood of developing AD. The DS brain has been typically described as developing relatively normally during the first few months postnatally ^5^, after which growth slows, with cortical areas being particularly reduced ^6^.

Yet underlying these group-level accounts are large individual differences at every level of description. We start with a consideration of individual differences in the genetics of DS and go on to examine studies of DS cell biology. We focus next on the broad individual differences in the DS brain, which recent studies have now identified as occurring as early as during fetal development. We go on to explore briefly some of the widespread individual differences in cognitive outcomes in DS, particularly with respect to language and memory, and challenge assumptions that individuals with DS are global rather than local processors ^7^. In the following section, we argue that individual differences in sleep patterns in DS are likely to be an important contributor to the differences in language, memory, and AD outcome. We then look briefly at mouse models of DS and AD. We finally conclude that a focus on individual differences at every level across the syndrome is likely to yield deeper insights into genotype/phenotype associations.

## Individual differences in Down syndrome genetics

The most common cause of DS is the additional copy of an entire chromosome 21. In ~88% of cases, the extra copy is maternally derived, through an error in cell division called non-disjunction. The extra chromosomal content can occur through different mechanisms and at different points during the formation of germ cells. Non-disjunction ^8^ can arise during meiosis I (~65% maternal; ~3% paternal), during meiosis II (~23% maternal; ~5% paternal), or from a mitotic error (~3%). DS can also occur when only a segment of chromosome 21 has three copies (partial trisomy) ^9^ or when the whole chromosome is triplicated but only a proportion of the cells are trisomic (mosaicism) with other cells being normal. Mosaicism is found in ~1.3–5% of cases ^10^, but it is possible that mosaicism occurs more frequently, the low percentage being due to ascertainment bias, especially in cases with low-level mosaicism. Further genetic differences can be introduced by variation in the amount of crossover during meiosis I. Research on parental origin or the mechanism of mosaicism is currently sparse, making it difficult to identify the main mechanism. While mosaicism has sometimes been claimed to yield a milder cognitive phenotype ^9, 11^, data addressing this are very sparse and, where they do exist, the degree of mosaicism does not correlate with phenotypic severity. Interestingly, though, mosaicism provides an excellent opportunity to study phenotypic differences, since disomic and trisomic cell lines derived from mosaics only differ in the extra chromosome 21 ^12^.

Translocation is another mechanism yielding DS, whereby some of the genetic material from chromosome 21, usually from the long arm, is moved to chromosome 14 or 22, or from the long to the short arm of chromosome 21. Translocation occurs in some ~4% of cases ^2, 13– 15^.

These multiple origins of DS need to be taken into account when considering differences between individuals with trisomy 21. Additionally, individual differences exist on other chromosomes. The euploid population, while free from gross chromosomal abnormalities, is nonetheless genetically different from one another, due to copy number variations (CNVs), single nucleotide polymorphisms (SNPs), and *de novo* mutations. Such differences also apply to people with DS, of course, who have many of these variants in addition to their extra copy of all or part of chromosome 21.

With full trisomy, intuitively it might be assumed that expression levels of triplicated genes are 1.5-fold that of the euploid population. However, this is not so. Gene expression is differentially regulated in different tissues, and each gene is subject to the potential of feedback control of expression levels. One recent study of whole genome expression in fibroblasts and lymphoblasts suggested that only a small majority of genes were over-expressed in the range predicted by gene dosage. In contrast, about a quarter showed no difference in expression between DS and diploid cells, and another quarter had intermediate expression ^16^. In a second study, also in lymphoblastoid cells, only 22% of the genes analyzed on chromosome 21 were actually over-expressed 1.5-fold ^17^. In this second study, a few were significantly more amplified (~7%), whereas, despite the three copies, many (>1/2) turned out to have near normal levels of expression, presumably due to compensatory mechanisms. It must be remembered that these studies were carried out in cell lines; the results may therefore not reflect the gene expression profiles of the cells from which they were derived and certainly will not represent the expression levels in other tissues. Interestingly, both of these cell studies additionally reported a considerable amount of inter-individual differences in gene expression. Expression studies are notoriously inconsistent. Nonetheless, however tentative the findings of the above two studies, it is clear that we cannot take for granted that an extra copy of chromosome 21 will result in a 1.5-fold increase in the level of gene expression. How irregular expression levels of triplicated genes on chromosome 21 (particularly those that may vary substantially between trisomic individuals), coupled with the heterogeneous origins, influence the DS neurocognitive phenotype remains an open but critical question.

Whilst the expression and role of individual genes are undoubtedly important, the genome-wide implications of trisomy 21 are too often neglected. Functionally, genes sit in a complex biological network. The breadth of influence of genes varies, but those involved in epigenetic mechanisms warrant special attention. Epigenetic mechanisms, including DNA methylation and post-translational histone modifications, contribute substantially to the regulation of gene expression across the genome, and so the effects of changes in epigenetic gene dosage are far-reaching. There are at least 11 genes and multiple microRNAs (miRNAs) on chromosome 21 that are involved in epigenetic mechanisms ^18^, including *DNMT3L* (a DNA methyltransferase), *DYRK1A* (a kinase), and *H2AFZP* (a histone variant). Relatively little research has gone into epigenetic processes in trisomy 21, although some studies indicate that people with DS have different DNA methylation from the euploid population ^19^. As mentioned above, in some of these genes, expression levels may vary between individuals (such as *BRWD1*, a transcriptional regulator). Trisomy 21 causes major disturbances in the level, activity, and subcellular localization of two major non-HSA21 transcription factors: *NFAT* ^20^ and *NRSF/REST* ^21, 22^. Both of these control the spatiotemporal expression patterns of thousands of downstream target genes, many of which are also transcription factors, generating a whole new layer of complexity. Individual differences in epigenetic regulation can of course also occur on genes not otherwise involved with chromosome 21, yielding potentially even wider individual differences in the mosaic DS population and those with DS arising from translocation.

One of the reasons why individuals with DS are at higher risk for AD than the general population is that the amyloid precursor protein ( *APP*) gene, implicated in the brain pathology of AD, lies on chromosome 21. Individuals with a translocation below the APP gene (i.e. without APP triplication) get DS but not AD. A number of genes that are functionally linked to *APP* are dysregulated in the DS brain, including *BACE2*, *APOE*, *CLU*, *PSEN1*, *PSEN2*, and *MAPT* ^22^. While amyloid pathology is necessary, triplication of APP alone is not sufficient to cause AD. Whereas many people with DS present with dementia in their 30s, even by age 70 or 80 some adults with DS do not have dementia despite their significant plaque pathology ^24, 25^.

Genes on other chromosomes also play an important role in AD and here, too, individual differences exist. The apolipoprotein gene ( *APOE*) on chromosome 19, also implicated in AD, harbors common variants: ε2, considered protective for AD (~7% of the general population); ε3, the most common allele (~79% frequency), neutral regarding AD risk; and ε4 (~14% frequency), thought to harbor the greatest risk for AD, particularly in carriers of two ε4 alleles. *APOE* variants modulate the age of onset of AD in DS ^26^. Interestingly, the distribution of the *APOE* polymorphisms differs across ethnicities, the above figures holding for Caucasians.

The effects of these *APOE* allelic differences are detectable early in life. A recent study of euploid babies between 2 and 25 months of age showed that those who carried the ε4 variant differed from non-carriers in their rate of myelin development, with ε4 carriers showing decreased growth in the mid and posterior brain regions ^27^. Similar allelic differences and their neural repercussions are likely also to occur in children with DS, impacting on other individual differences.

Other genes, e.g. *DYRK1A* and *RCAN1*, located on chromosome 21, have been shown to be functionally important in the pathogenesis of DS and AD when expression is increased ^28, 29^. Individual ethnic differences also matter. Indeed, the common variants of these genes are not significantly associated with AD in Caucasians, but there is some suggestion of an association of the *RCAN1* polymorphism in a small Chinese cohort ^30^. Other research has suggested that *BACE2* alleles, also located on chromosome 21, are important in AD, also affecting the age of dementia onset in DS ^31, 32^.

## Individual differences in Down syndrome cell biology

The advent of human induced pluripotent stem cells (iPSCs) has added an exciting new tool for understanding individual differences in DS and their relationship to AD ^12, 33^. Shi *et al.* ^33^ found that cortical neurons generated from iPSCs and embryonic stem cells from patients with DS developed AD pathologies in the form of insoluble intracellular and extracellular amyloid aggregates over months in culture, rather than years *in vivo*. Hyperphosphorylated tau protein, a hallmark of AD, was also localized to cell bodies and dendrites in iPS-derived cortical neurons from the patients with DS, recapitulating later stages of the AD pathogenic process. Interestingly, the same research group showed growth of amyloid-β plaques in iPSCs grown from tissue from a DS infant as young as 17 months ^33^, attesting to the developmental nature of the brain pathology. Furthermore, an isogenic iPSC model of DS derived from a 16 year old with mosaic DS ^12^ recapitulated these AD-related phenotypes and demonstrated that neurons from trisomy 21 iPSCs accumulate DNA double-strand breaks much faster than those from isogenic euploid controls. It is currently not known whether, but it is assumed that, such accumulated DNA damage is randomly distributed in the genome and as such may increase the variability of pathological phenotypes on the cellular level ^12^.

## Individual differences in Down syndrome brains

As mentioned, it used to be thought that the DS brain developed relatively normally throughout fetal life and during the first months postnatally ^5^. This assumption has turned out to be incorrect. New studies reveal that DS prenatal brain size is only relatively normal until about 20–24 weeks gestation, after which individual differences in fetal brain development emerge (unpublished data, Rutherford & Patkee 2015). Some DS brains show reduced volume of the hippocampus, cerebellum, and occipital-frontal areas already during fetal life. In some DS brains, there is initially more or less normal dendritic formation and arborization, but this is followed by a stagnation in the developmental process; subsequently dendrites increase neither in number nor in complexity as the DS fetus develops ^34^. At birth, many DS brains already have smaller dendritic arborization ^35– 37^ and fewer synapses ^38, 39^, likely to contribute to the reduced functional brain connectivity found in many newborns with DS ^40^.

Despite large individual differences, some DS brains are difficult to distinguish from the neurotypical case during fetal development (unpublished data, Rutherford & Patkee 2015), but the neural phenotype becomes progressively more pronounced in DS as development proceeds, with increasing dissociations between cortical thickness (increased) and surface area (reduced) in, for example, frontal and temporal regions ^41^. However, yet again, individual differences are apparent, particularly in the early stages of development. In other words, individual differences at both the structural and the functional levels can start very early in the DS developmental trajectory, subsequently yielding large individual differences in functional connectivity, which correlate, for instance, with communication skills ^42^. Finally, 40% of infants with DS are born with congenital heart disease, which also potentially compromises blood flow to the brain, but even those without heart problems ultimately go on to develop atypical brains ^43^.

Examining the brains of adults with DS, MRI studies have demonstrated that the size of the cerebellum, hippocampus, and cortex is significantly smaller than in the neurotypical case, while basal ganglia are similar in size and ventricles are enlarged ^44^. Individual differences are particularly apparent when comparing DS adults with or without dementia; the former have reduced ventricular, hippocampal, and caudate volumes, as well as increased levels of peripheral cerebrospinal fluid (CSF), compared to those without dementia ^45^. The differences between those with and without dementia can start very early *. In vivo* studies of children with DS identified plaques in DS brains as early as 8 years of age ^46^. To be noted, however, were the large individual differences, with some DS brains having no plaques until early adulthood.

## Individual differences in Down syndrome cognition

Atypical cognitive phenotypes in DS become increasingly evident across the lifespan ^47^. Children under 12 months old often show few cognitive differences from neurotypical controls on standardized tests (due, perhaps, to a lack of sensitivity to detect them) but, as they get older, the rate of intellectual development in DS slows considerably.

Most of the cognitive studies of DS have reported group data, comparing DS either to neurotypical controls ^48– 50^ or to other neurodevelopmental disorders ^51– 56^. Yet hidden within these group data are wide individual differences, particularly in IQ scores, language, and other measures ^57– 61^. And these differences start early; in our recent research on infants/toddlers with DS, standard composite scores (not dissimilar to IQ scores) on the Mullen Scales of Early Learning ^62^ show significant variation, with many young children scoring at floor, while some others’ scores reach the 80s to 90s. In adults with DS, some 50% have IQs at floor, whereas a few have IQs in the 70s or above ^63^. The significance of these individual differences is being increasingly recognized, such that we are developing new task batteries to detect the wide range of scores more precisely ^64, 65^.

Individual differences in basic-level processes like reaction time, attention, and memory impact on developmental trajectories over time. For example, the DS memory profile is associated with poor short-term verbal memory ^66^ and poor long-term visual memory ^54^. In contrast, implicit memory is thought to be comparable to age-matched neurotypicals ^49^. However, again these observations are based on group data, with individual memory profiles being significantly more variable ^67^. In Vicari *et al.*’s ^49^ paper, implicit memory—measured by reaction time—was on average longer in DS than in neurotypicals, but the standard deviations were almost three times larger in the DS group. This might mean that some individuals with DS had shorter reaction times even than the controls. Such individual differences are camouflaged when reporting average group data yet are critical to fully understand the DS phenotype.

As mentioned in the introduction, DS is often described as having better visuospatial memory than verbal memory, as well as better global processing than local processing. First, individual differences are large, and, second, in-depth probing of processing across modalities (visual/auditory) and across levels of processing (low-level perceptual processes vs. high-level) yielded no consistent global processing style ^7^.

 Another domain that yields wide individual differences in DS is language—for some, considered the domain of greatest vulnerability in the syndrome ^59, 68^. This claim is made from comparisons of children with DS to neurotypicals at the group level. A very different picture emerges when individual differences are taken into consideration. For example, Zampini and D’Odorico ^69^ reported that, in their longitudinal study of DS vocabulary acquisition, at 36 months the lowest scoring child was nonverbal, while the highest scoring child was close to the normal range, producing 243 words. When the same children were assessed 6 months later, the nonverbal child remained nonverbal, whereas the one with the most developed language had doubled production to nearly 500 words. This highlights the wide individual differences in DS language development, which persists into adulthood ^70^.

However, in order to fully understand how those with DS develop, it is crucial to study how individual differences in underlying processes (e.g., auditory/visual attention, motor control) constrain higher-level cognitive outcomes (e.g. language). For example, there is much greater variability in the timing of the onset of muscle activation in DS than in neurotypicals ^71, 72^, such that distal muscles are often activated before proximal muscles. It is possible, then, that the variability in underlying mechanisms, such as muscle activation, becomes subsequently measurable as differences in DS cognitive abilities.

Another example from our recent work on very young children with DS reveals that individual differences in an electrophysiological measure of auditory attention in toddlers with DS are associated with differences in language ability ^73^. On average, the toddlers with DS oriented to changes in pitch more than changes in speech, but wide individual differences emerged: toddlers with DS who oriented more to changes in pitch had worse expressive language, suggesting that those who rely excessively on global properties of sounds (e.g., tone, changes in pitch) are not using an optimal strategy for language learning. As a group, the toddlers were also slow at disengaging attention from visual stimuli, but again individual differences indicated that those who were particularly poor at disengaging visual attention had worse language ability.

Thus, individual differences in both visual and auditory attention predict language differences in the DS children, indicating that small differences in attention during very early development impact the subsequent development of other higher-level domains like language ^74^.

## Individual differences in Down syndrome sleep

Sleep has a crucial function in ensuring metabolic homeostasis and the clearance of toxins like β-amyloid from the brain. Using real-time assessments of tetramethylammonium diffusion and two-photon imaging in live mice, Xie and colleagues ^75^ showed that deep sleep is associated with a 60% increase in the interstitial space, resulting in a striking increase in convective exchange of CSF with interstitial fluid. The researchers showed that convective fluxes of interstitial fluid increase the rate of β-amyloid clearance during sleep. The restorative function of sleep may thus be a consequence of the enhanced removal of potentially neurotoxic waste products that accumulate in the central nervous system when awake. Therefore, if individuals with DS show differences in their sleep architecture, such β-amyloid clearance may be differentially compromised.

Indeed, there is an increased risk of sleep fragmentation in DS because of obstructive sleep apnea in this population ^76– 79^. Edgin and collaborators found that children with DS with obstructive sleep apnea syndrome had impaired executive function as well as verbal IQs nine points lower than those without apnea ^79^. Even in the euploid population, poor sleep quality, particularly sleep fragmentation, is a strong predictor of lower academic performance ^80^, reduced attentional capacities ^81^, poor executive function ^82^, and challenging behaviors ^83^. As far as young adults with DS are concerned, our ongoing work suggests that 16–35 year olds with disturbed sleep have poorer cognitive scores, lower adaptive behavior scores, and poorer verbal fluency ^65^. Again, individual differences in sleep patterns start early. Our current work with infants and toddlers with DS is revealing correlations between increased sleep fragmentation (not duration) and decreased memory, language, and attention shifts ^84^. If amyloid clearance is subject to wide individual differences in DS due to varying levels of sleep fragmentation, this may be a clue to one of the reasons why some individuals go on to present with dementia and others do not. It is therefore possible that individual differences in sleep patterns in the DS population across the lifespan, together with other factors, impact on risk and protective factors for AD.

## Individual differences in Down syndrome animal models

Murine models of DS and of AD-DS exist, based on ortholog genes to human chromosome 21, which are located on chromosomes 10, 16, and 17 in the mouse ^85, 86^. Most are kept on inbred, identical genetic backgrounds and are used to identify genes associated with neurobehavioral traits. Although rarely reported, it is clear that, even in inbred strains, phenotypic variability occurs in terms of rate of development, disease, and behavioral traits. Using prenatal and postnatal cross-fostering methods, several studies have shown that these individual differences stem from environmental factors, such as amount of maternal licking/grooming, i.e. epigenetic programming by maternal behavior ^87^, rather than genetic differences between offspring ^88– 90^. It is becoming increasingly likely that individual epigenetic changes arising from experience of a parent can be transmitted to their offspring and to future generations ^91^. Variations in rat maternal care have been shown to affect hippocampal function as well as performance on hippocampal-dependent learning and memory tests in the offspring ^88^. There is every reason to believe that mouse models of DS would reveal similar effects (see discussion in 86). For instance, transgenic mice overexpressing *Dyrk1A*, a candidate gene on chromosome 21, show serious alterations in adult neurogenesis, including reduced cell proliferation rate, altered cell cycle progression, and reduced cell cycle exit, leading to premature migration, differentiation, and reduced survival of newly born cells. In addition, less proportion of newborn hippocampal *TgDyrk1A* neurons are activated upon learning, suggesting reduced integration in learning circuits. A number of these alterations can be normalized both pharmacologically and by environmental stimulation ^92^.

## Concluding thoughts

The fact that DS presents with so many individual differences, at so many levels, clearly indicates that thinking of DS merely in terms of an extra copy of chromosome 21 would be simplistic. Many other genetic, epigenetic, and environmental factors play a role in how the DS phenotype expresses itself in each individual. Whereas mosaicism has sometimes been claimed to yield a milder cognitive phenotype, albeit with few data to support the claim, it remains unknown whether genetic differences in the original, individual causes of DS lead to corresponding differences in neurocognitive outcomes. Numerous other interacting factors are likely to contribute to individual differences and cognitive-level outcomes in DS, including early neural development, sleep, attention, memory, and the environment.

It is also important to note that having a neurodevelopmental disorder like DS actually changes the environment (both social and physical) in which infants and children develop, in terms of parental expectations and their interactions with their child ^61, 93^. A more complex, dynamic view is thus required of how individual differences in the child’s social, cultural, and physical environments interact with individual differences in genetics and epigenetics.

One thing is clear: scientists cannot consider those with DS as a homogeneous group. Consideration of individual variation at multiple levels opens a series of new questions raised in this review that remained hidden in studies at the DS group level. Thus, scientists must take on board the crucial importance of individual differences if we are to understand fully the relationships between genotype and the emerging phenotype, and why some individuals with DS do not go on to present with dementia despite their brain histopathology. Moreover, it is becoming increasingly clear that Alzheimer’s dementia is a *developmental* disease ^74^ and that trisomy 21 is a particularly good model for understanding many of the complexities of that developmental process across the lifespan.

## Abbreviations

AD            Alzheimer’s disease

APOE       Apolipoprotein

APP          Amyloid precursor protein

CSF          Cerebrospinal fluid

DS            Down syndrome

iPSCs       Induced pluripotent stem cells
